# Phylogenetic relationships of some species of the family Echinostomatidae Odner, 1910 (Trematoda), inferred from nuclear rDNA sequences and karyological analysis

**DOI:** 10.3897/CompCytogen.v9i2.4846

**Published:** 2015-06-03

**Authors:** Gražina Stanevičiūtė, Virmantas Stunžėnas, Romualda Petkevičiūtė

**Affiliations:** 1 Institute of Ecology of Nature Research Centre, Akademijos str. 2, LT–08412 Vilnius, Lithuania

**Keywords:** *Echinochasmus*, *Stephanoprora*, Echinostomatidae, karyotype evolution, intermediate host, rDNA, ITS2, 28S

## Abstract

The family Echinostomatidae Looss, 1899 exhibits a substantial taxonomic diversity, morphological criteria adopted by different authors have resulted in its subdivision into an impressive number of subfamilies. The status of the subfamily Echinochasminae Odhner, 1910 was changed in various classifications. Genetic characteristics and phylogenetic analysis of four Echinostomatidae species – *Echinochasmus* sp., *Echinochasmus coaxatus* Dietz, 1909, *Stephanoprora pseudoechinata* (Olsson, 1876) and *Echinoparyphium mordwilkoi* Skrjabin, 1915 were obtained to understand well enough the homogeneity of the Echinochasminae and phylogenetic relationships within the Echinostomatidae. Chromosome set and nuclear rDNA (ITS2 and 28S) sequences of parthenites of *Echinochasmus* sp. were studied. The karyotype of this species (2n=20, one pair of large bi-armed chromosomes and others are smaller-sized, mainly one-armed, chromosomes) differed from that previously described for two other representatives of the Echinochasminae, *Echinochasmus beleocephalus* (von Linstow, 1893), 2n=14, and *Episthmium bursicola* (Creplin, 1937), 2n=18. In phylogenetic trees based on ITS2 and 28S datasets, a well-supported subclade with *Echinochasmus* sp. and *Stephanoprora pseudoechinata* clustered with one well-supported clade together with *Echinochasmus japonicus* Tanabe, 1926 (data only for 28S) and *Echinochasmus coaxatus*. These results supported close phylogenetic relationships between *Echinochasmus* Dietz, 1909 and *Stephanoprora* Odhner, 1902. Phylogenetic analysis revealed a clear separation of related species of Echinostomatoidea restricted to prosobranch snails as first intermediate hosts, from other species of Echinostomatidae and Psilostomidae, developing in Lymnaeoidea snails as first intermediate hosts. According to the data based on rDNA phylogeny, it was supposed that evolution of parasitic flukes linked with first intermediate hosts. Digeneans parasitizing prosobranch snails showed higher dynamic of karyotype evolution provided by different chromosomal rearrangements including Robertsonian translocations and pericentric inversions than more stable karyotype of digenean worms parasitizing lymnaeoid pulmonate snails.

## Introduction

The family Echinostomatidae Looss, 1899 is a heterogeneous group of cosmopolitan, hermaphroditic digeneans. Adult echinostomatids are predominantly found in birds, and also parasitize mammals including man, and occasionally reptiles and fishes (Huffman and Fried 1990, Kostadinova and Gibson 2000, Kostadinova 2005a). Morphological diversity of this group and/or the diversity of the criteria adopted by different authors have resulted in its subdivision into an impressive number of subfamilies (Kostadinova and Gibson 2000). The Echinostomatidae has been viewed as a monophyletic taxon, with some exceptions, but some authors suggested that the family Echinostomatidae is polyphyletic and elevated the Echinochasminae Odhner, 1910 to full family rank (Odening 1963, Sudarikov and Karmanova 1977). Kostadinova (2005a) accomplished the last revision of the Echinostomatidae accepting 11 subfamilies and 44 genera after the vast comparative morphological study based on the examination of type and freshly collected material, and a critical evaluation of published data. Afterward, she retained the subfamilial status of the Echinochasminae with similar composition to that proposed in 1971 by Yamaguti.

The karyotypes of more than 20 species of the subfamily Echinostomatinae Looss, 1899 belonging to the genera *Echinostoma* Rudolphi, 1809, *Echinopharyphium* Dietz, 1909, *Hypoderaeum* Dietz, 1909, *Neoacanthoparyphium* Yamaguti, 1958, *Moliniella* Hübner, 1939, and *Isthmiophora* Lühe, 1909 have been described; most species had 2n=20 or 2n=22, except some species (for review, see Baršienė 1993). The karyotypes of two species of the subfamily Echinochasminae, namely *Echinochasmus beleocephalus* (von Linstow, 1893), 2n=14, and *Episthmium bursicola* (Creplin, 1937), 2n=18, have been reported by Baršienė and Kiselienė (1990).

The use of molecular approaches to determine phylogenetic relationships of digeneans has grown very rapidly since 1990s and molecular-based studies on echinostomes have been carried out to date (Morgan and Blair 1995, 1998a, 1998b, 2000, Petrie et al. 1996, Grabda-Kazubska et al. 1998, Kostadinova et al. 2003, Saijuntha et al. 2011, Georgieva et al. 2013, 2014, Noikong et al. 2014, Selbach et al. 2014, Kudlai et al. 2015). The genus *Echinochasmus* Dietz, 1909 (as well as *Echinostoma* and *Echinopharyphium*) is one of the most species–rich genera in Echinostomatidae (Kostadinova and Gibson 2000); however, no one species of this genus was involved in molecular phylogenetic studies of the Digenea (Cribb et al. 2001, Olson et al. 2003, Olson and Tkach 2005).

The present study is mainly focused on comparative analysis of species belonging to the subfamily Echinochasminae. Two regions of rDNA, ITS2 and partial 28S, and karyotype of cercaria of *Echinochasmus* sp., parasite of the gravel snail *Lithoglyphus naticoides* (C. Pfeiffer, 1828) are presented there as well as DNA sequences of adult specimen of type-species of *Echinochasmus*, *Echinochasmus coaxatus* Dietz, 1909 from the final host *Podiceps nigricollis* C. L. Brehm, 1831. Morphology of the *Echinochasmus* sp. cercaria from the same population of *Lithoglyphus naticoides* was previously described by Stanevičiūtė et al. (2008).

## Materials and methods

The digeneans for this study were obtained from naturally infected hosts. Seven specimens of gravel snail *Lithoglyphus naticoides* infected with parthenites of *Echinochasmus* sp. were collected at water reservoir of the dammed up River Nemunas near Kaunas in Lithuania (54°51.38'N, 24°09.08 E’). The specimens of snail *Valvata piscinalis* (Müller, 1774) infected with parthenites of *Echinoparyphium mordwilkoi* Skrjabin, 1915 were collected from the River Ūla, Lithuania (54°7.76'N, 24°27.76'E). The ethanol fixed adult specimen of *Echinochasmus coaxatus* recovered from *Podiceps nigricollis* in Kherson region (Ukraine) was received from collection of Department of Parasitology, I.I. Schmalhausen Institute of Zoology of NAS of Ukraine. Adult trematodes from *Larus melanocephalus* (Temminck, 1820) and cercariae from *Hydrobia acuta* (Draparnaud, 1805) were described as *Stephanoprora pseudoechinata* (Olsson, 1876) by Kudlai and Stunžėnas (2013); rDNA sequences of these specimens were used for comparative analysis in this study.

Living *Lithoglyphus naticoides* snails were incubated in 0.01% colchicine in well water for 12–14 h at room temperature and afterward, dissected. The infected tissues from crushed snails were transferred to distilled water for 40–50 min and fixed in a freshly prepared Carnoy’s solution I (Farmer’s solution) composed of 3 parts of 95% ethanol and 1 part glacial acetic acid. Chromosome slides were prepared using air-dried method and analysed after conventional Giemsa staining (Petkevičiūtė and Stanevičiūtė 1999). The karyotypes were constructed by arranging the chromosome pairs in order of decreasing size. Chromosomes of 11 high quality metaphase plates were measured using Image-Pro Plus v3 software. Chromosome measurements included length of individual chromosomes, relative length, and centromeric index. These parameters were used for description of chromosome morphotype according to standard nomenclature of Levan et al. (1964). Data were analyzed using the Student’s *t* test. Results were considered significant when P<0.05. The same nomenclature was applied to the karyotype of the other seven species used for comparison: *Episthmium bursicola*, *Echinochasmus beleocephalus*, *Echinopharyphium aconiatum* Dietz, 1909, *Istmiophora melis* (Schrank, 1788) Lühe, 1909, *Hypoderaeum conoideum* (Bloch, 1782), *Sphaeridiotrema globulus* (Rudolphi, 1814), and *Echinostoma revolutum* (Fröelich, 1802) Looss, 1899. Karyotypic data of these taxa were obtained from Baršienė and Kiselienė (1990), Baršienė (1993) and Mutafova (2001).

The DNA extraction (without proteinase or lysis buffer treatment) was performed in sterile Tris-borate-EDTA (TBE) buffer. In previous study this method allowed us to extract high quality DNA from tissue of molluscs (Stunžėnas et al. 2011) and trematodes (Petkevičiūtė et al. 2014). An entire nuclear 5.8S-ITS2-28S DNA sequence of ribosomal DNA (~460bps: 5.8S ribosomal RNA gene, partial sequence; internal transcribed spacer 2, complete sequence; and 28S ribosomal RNA gene, partial sequence) was amplified using primers: 3S (5’- CGG TGG ATC ACT CGG CTC GTG -3’), forward direction; 28S (5’- CCT GGT TAG TTT CTT TTC CTC CGC -3’), reverse direction (Bowles et al. 1995). The 5’ end of the 28S rRNA gene sequence (~1,200 bps), not overlapping with the previous sequence, was amplified using two primers: Digl2 (5’- AAG CAT ATC ACT AAG CGG -3’) forward direction; L0 (5’- GCT ATC CTG AG(AG) GAA ACT TCG-3’) reverse (Tkach et al. 1999). DNA fragments were amplified via a standard Polymerase Chain Reaction (PCR) according to Petkevičiūtė et al. (2014).

DNA sequences of representative species of the superfamily Echinostomatoidea and outgroup taxa were downloaded from GenBank and included in the phylogenetic analysis and/or pairwise sequence comparisons together with our data. For phylogenetic analyses the sequences were aligned with ClustalW (Thompson et al. 1994) with an open gap penalty of 15, and a gap extension penalty of 6.66. For data sets we estimated the best-fit model of sequence evolution using jModeltest v. 0.1.1 software (Posada 2008). Neighbour-joining (NJ) (Saitou and Nei 1987), maximum parsimony (MP) (Nei and Kumar 2000) and maximum likelihood (ML) phylogenetic trees were obtained and analysed using MEGA 5 (Tamura et al. 2011). Supports to internal branches for the trees were estimated by bootstrap analyses with 1000 replicates. The genetic distances of neighbour joining tree were calculated by Tamura-Nei (Tamura and Nei 1993) for 28S gene and 5.8S-ITS2-28S rDNA region datasets. Maximum likelihood trees were obtained using general time reversible model with a gamma distribution of rates and a proportion of invariant sites (GTR+G+I) for the both datasets. Gamma shape and number of invariant sites were estimated from the data. Parsimony analysis based on subtree pruning and regrafting (SPR) was used with default parsimony settings.

## Results

### Karyotype of *Echinochasmus* sp.

**Figure 1.**
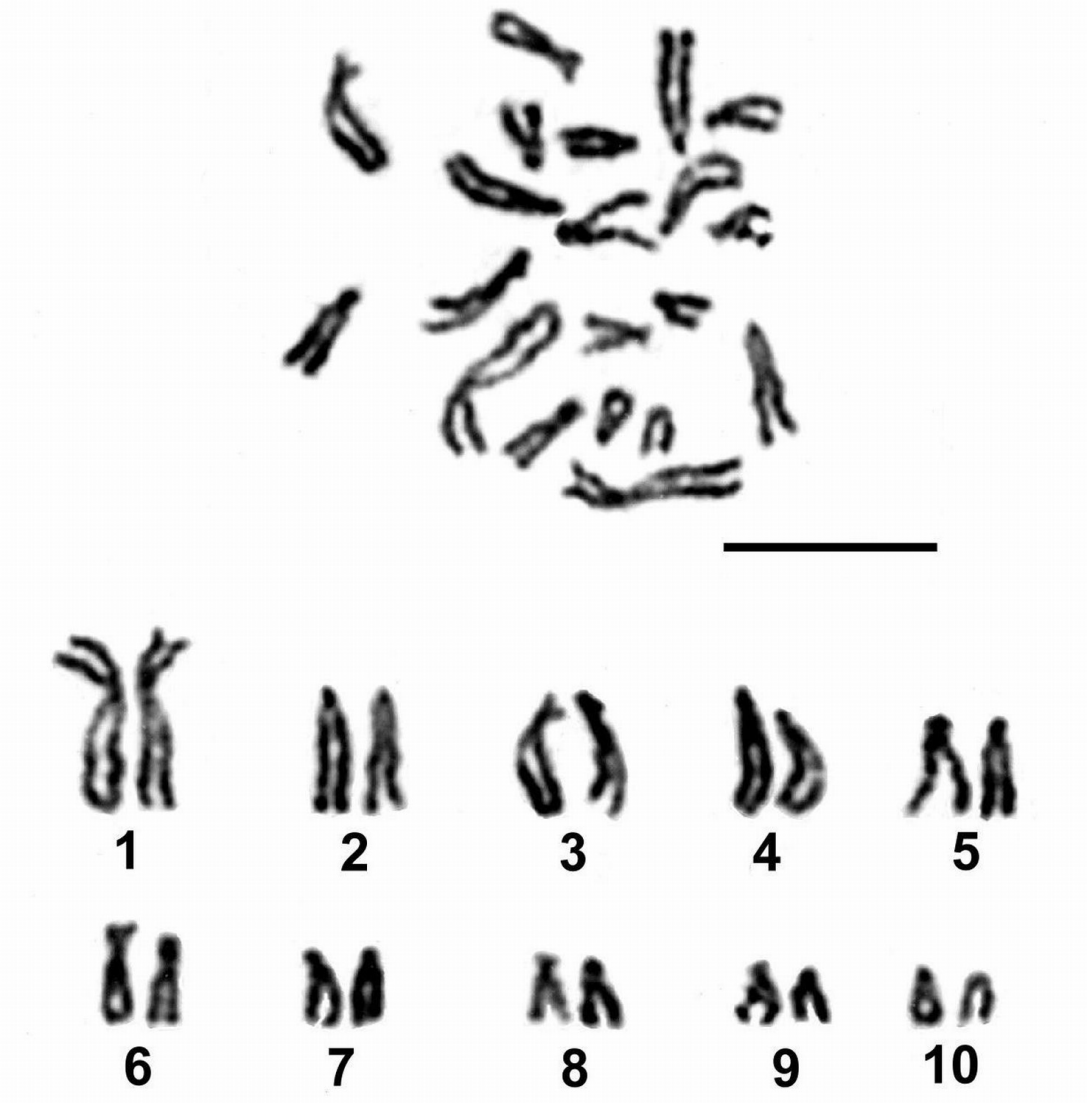
Mitotic metaphase and karyotype of *Echinochasmus* sp. Bar = 10 µm.

Chromosomes of 113 mitotic metaphase spreads from three molluscs revealed that karyotype of *Echinochasmus* sp. is 2n=20; it consists of one pair of large chromosomes and nine pairs of smaller-size chromosomes. Also, the percentage of aneuploid cells (2n=18–19) was 10.62%. Twelve spreads displaying values lower than modal, represent aneuploidies or (more likely) loss of chromosomes during processing, a technical artefact commonly encountered with the slide preparation method used. The measurements of mitotic chromosomes showed ten chromosome pairs ranging in size from 2.11 to 7.64 μm (Fig. 1, Table 1). The mean total length of the haploid complement is 40.07 μm. The homologues of the 1^st^ pair are significantly large than the remaining chromosomes and comprise about 19% of the total chromosome complement length. According to the centomeric index value they are of submeta-or metacentrics. The remaining chromosomes decrease in size fairly gradually. Three pairs (2^nd^, 4^th^ and 5^th^) fall into an intermediate position between acrocentric and subtelocentric; pair 3^rd^ is subtelocentric - submetacentric; pair 6^th^ is submetacentric and four last chromosome pairs (7^th^ – 10^th^) are subtelocentric.

**Table 1.** Morphometric analysis of chromosomes of *Echinochasmus* sp. Stanevičiūtė, Petkevičiūtė & Kiselienė, 2008.

Chromosome number	Absolute length (mm)	Relative length (%)	Centromeric index	Classification
1	7.64*±1.69	18.97±1.61	37.45±1.64	sm-m
2	4.99±0.79	12.51±0.68	10.44± 2.66	a-st
3	4.72±0.98	11.73±0.66	23.64±2.25	st-sm
4	4.46±0.88	11.09±0.58	14.18±3.62	st-a
5	3.98±0.78	9.89±0.60	13.95±4.13	st-a
6	3.69±0.63	9.23±0.64	30.39±5.27	sm
7	3.16±0.53	7.89±0.41	20.71±2.82	st
8	2.81±0.40	7.05±0.44	19.41±2.93	st
9	2.51±0.28	6.33±0.46	22.92±5.25	st
10	2.11±0.38	5.29±0.71	19.17±4.32	st

* - mean±SD; m - metacentric; sm - submetacentric, st - subtelocentric; a - acrocentric chromosomes

### Molecular analysis

**Figure 2.**
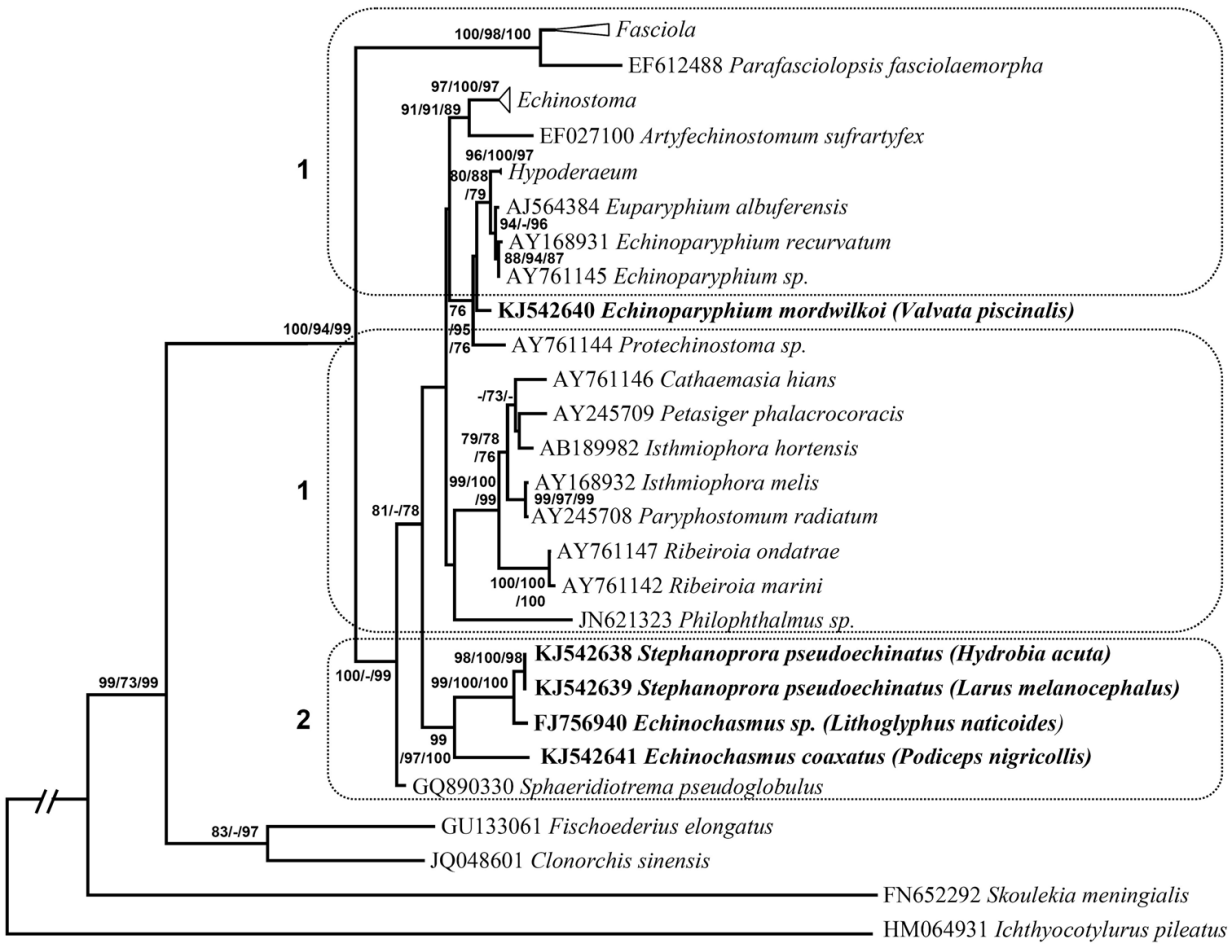
Phylogenetic ITS2 tree. Maximum likelihood phylogenetic tree based on analysis of ribosomal DNA sequences (5.8S-ITS2-28S). Bootstrap percentages refer to maximum likelihood / neighbor-joing / maximum parsimony analysis. Only bootstrap values above 70% are shown. GenBank accession numbers are indicated before species names. Names of the target species are in bold; their hosts are presented in parentheses. Compressed clades: *Fasciola* (comprised sequences under GenBank accession numbers AM900370, EF534995, EF612486, JF496715), *Echinostoma* (AF067850, AF067852, AJ564383, AY168930, EPU58100, ETU58097, ELU58099, GQ463131, GQ463132), *Hypoderaeum* (AJ564385, GQ463134). Dotted rectangles 1 indicate digeneans whose life cycles include Lymnaeoidea as first intermediate host; dotted rectangle 2 indicates digeneans whose life cycles include prosobranch snails as first intermediate hosts.

**Figure 3.**
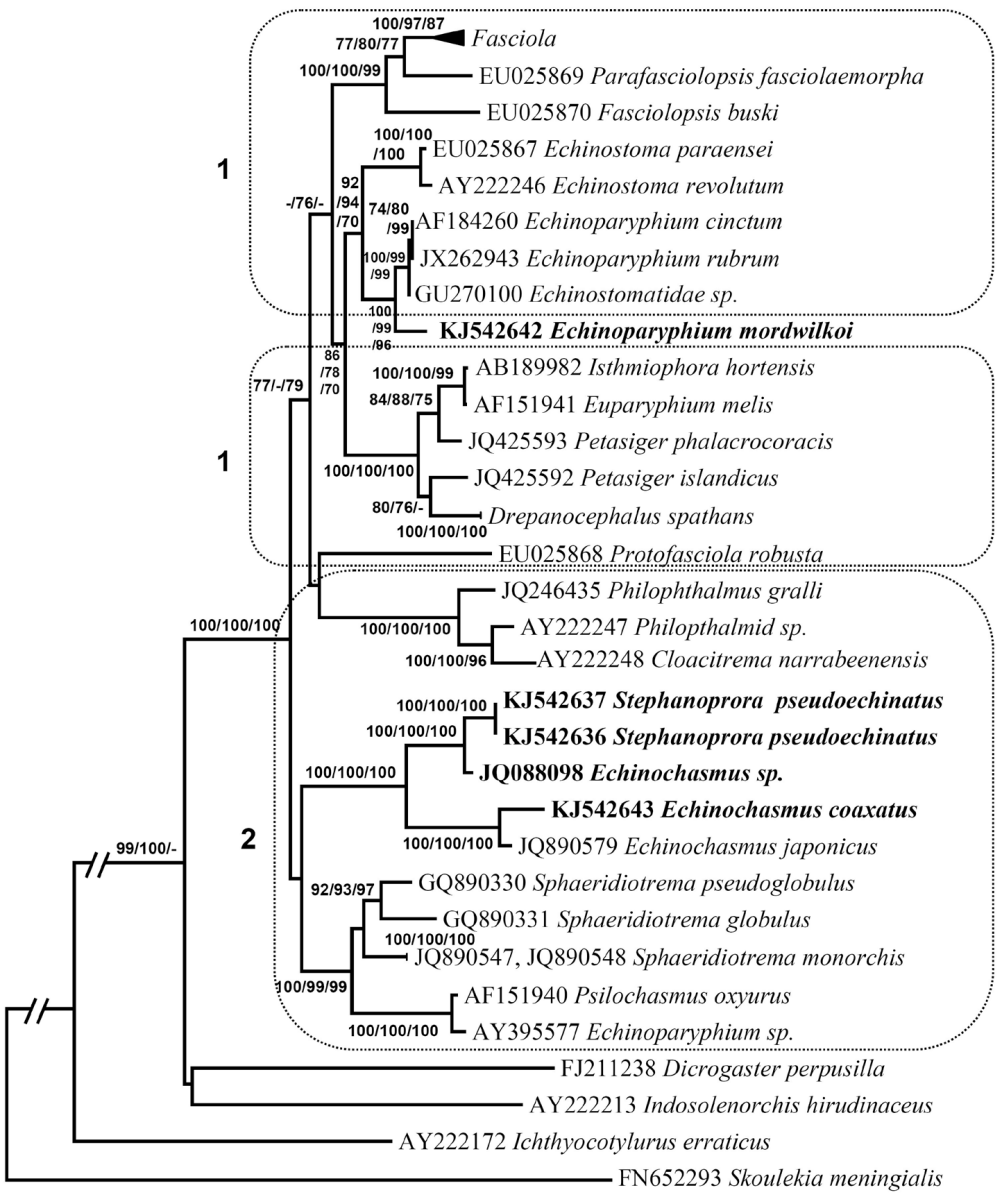
Phylogenetic 28S tree. Maximum likelihood phylogenetic tree based on analysis of ribosomal 28S gene DNA partial sequences. Bootstrap percentages refer to maximum likelihood / neighbor-joing / maximum parsimony analysis. Only bootstrap values above 70% are shown. GenBank accession numbers are indicated before species names. Names of the target species are in bold.Compressed clade *Fasciola* comprised sequences under GenBank accession numbers AY222244, EU025871, EU025872, HM004190). Dotted rectangles 1 indicate digeneans whose life cycles include Lymnaeoidea as first intermediate host; dotted rectangle 2 indicates digeneans whose life cycles include prosobranch snails as first intermediate hosts.

New sequences from two different regions of nuclear ribosomal DNA were obtained: the 5.8S-ITS2-28S and the 5’ end of the 28S gene, which does not overlap with the previous sequence. Complete nucleotide sequences are available in GenBank (Figs 2, 3). Pairwise comparisons of newly obtained sequences demonstrated that *Echinochasmus* sp. was closest to *Stephanoprora pseudoechinata*. These sequences of *Echinochasmus* sp. differed from sequences of *Stephanoprora pseudoechinata* by 12 out of 653 base pairs (1.84%) in the 5.8S-ITS2-28S region and by 15 out of 1070 base pairs (1.4%) in the sequenced portion of the 28S gene. All other differences among the new sequences were more significant, sequence divergence ranged from 13.59 to 23.15% in the 5.8S-ITS2-28S region and from 6.5 to 10.76% in the portion of the 28S gene. Blast searches (http://www.ncbi.nlm.nih.gov/blast/Blast.cgi) performed on these sequences demonstrated the highest matches with sequences of digenean trematodes of superfamily Echinostomatoidea. The new sequences were aligned with sequences of representative species of this superfamily. The aligned dataset of the 5.8S-ITS2-28S rDNA region included 35 sequences of the Echinostomatoidea and 408 sites after trimming the ends to match the shortest aligned sequences. This alignment without outgroups showed a high sequence divergence of ITS2 rDNA region and comprises 228 variable (56%) and 175 (43%) parsimony informative sites. The aligned dataset of the partial 28S gene included 33 sequences of the Echinostomatoidea and was comprised of 990 sites after trimming the ends to match the shortest aligned sequences. This alignment without outgroups comprises 341 variable (34.44%) and 250 (25.25%) parsimony informative sites.

Maximum likelihood, neighbor-joining and maximum parsimony analyses of these sequences, including representative species of superfamily Echinostomatoidea, produced identical topology of phylogenetic trees (Figs 2, 3). The *Echinochasmus* sp. Stanevičiūtė et al. 2008 clustered together with *Stephanoprora pseudoechinata* in a 94–100% supported subclade in the ITS2 phylogenetic tree (Fig. 2) and a 100% supported clade in the 28S phylogenetic tree (Fig. 3). This subclade clustered together with other species from *Echinochasmus* genus and formed a well-supported monophyletic clade, clearly separated from clades containing other species of Echinostomatoidea families. *Echinoparyphium mordwilkoi* clustered in a 96–100% supported clade with *Echinoparyphium* spp. Species of these genera formed a 99–100% supported subclade without separate branch of *Echinoparyphium mordwilkoi* (Fig. 3).

## Discussion

Sequence divergence between *Stephanoprora pseudoechinata* and *Echinochasmus* sp., 1.84% in the 5.8S-ITS2-28S rDNA region and 1.4% in the partial 28S gene, falls within the level of intragenus variability. Both taxa made up a strongly supported clade together with the type-species of the genus *Echinochasmus*, *Echinochasmus coaxatus*. These results imply that macrocercous cercaria of *Echinochasmus* sp. may be attributed to the genus *Stephanoprora* Odhner, 1902. According to Kostadinova (2005a), data on the life histories of some Echinochasminae species (including, probably, *Echinochasmus macrocaudatus* Ditrich, Scholz & VargasVazques, 1996) tend to support the affiliation of species to *Stephanoprora* rather than to *Echinochasmus* on the presence of a long-tailed cercarial stage. On the other hand, *Stephanoprora pseudoechinata* is a marine species, while *Echinochasmus* sp. Stanevičiūtė et al. 2008 is a parasite of freshwater organisms, a finding that shows a considerable ecological plasticity in this group. Sudarikov and Karmanova (1977) stated that the ontogenetic character state of Echinochasminae species concerning the absence of well-developed collar with collar spines in the morphology of cercaria, indicates that echinochasmids is a more ancient group than other echinostomatids. The phylogenetic relationships estimated by ITS2 and 28S sequences partly support this hypothesis, because *Echinochasmus* sp. Stanevičiūtė et al. 2008 and *Stephanoprora pseudoechinata* were clustered in one clade with *Sphaeridiotrema globulus* (Psilostomidae) in the 28S tree. Cribb et al. (2001) stated that from 144 known life cycles of Echinostomatidae species about two-thirds of the first intermediate hosts are lymnaeoid pulmonates but there are also significant numbers of species developing in prosobranchs. Ecological preferences of Echinostomatidae species suggest that there has been a strong co-evolution with the Lymnaeoidea and a less frequent association with a few prosobranch taxa. On the contrary, all 18 species of *Echinochasmus* with known life cycles are restricted to prosobranchs. *Echinoparyphium mordwilkoi*, that shows a separate position from *Echinochasmus* in the molecular analyses (Figs 2, 3), is restricted to the lower heterobranch *Valvata piscinalis* (Valvatoidea). Most of Psilostomidae species also admit for the first intermediate host a prosobranch snail (Grabda-Kazubska et al. 1991), except those ones belonging to the genus *Ribeiroia* Travastos, 1939, which position in this family is questionable (Wilson et al. 2005). The species of this genus originally have parasitized pulmonate snails. In the 28S phylogenetic tree, the clade uniting *Echinochasmus* spp. and *Stephanoprora* sp. clustered with Psilostomidae (*Psilochasmus oxyurus* (Creplin, 1825) and *Sphaeridiotrema globulus*), whose life cycles include prosobranch snails as first intermediate host. The isolate of redia gathered from the prosobranch snail *Gabbia vertiginosa* (Frauenfeld, 1862), despite being identified as *Echinoparyphium* sp. (unpublished data from Genbank), also clustered with *Psilochasmus oxyurus* and *Sphaeridiotrema globulus*. Grabda-Kazubska et al. (1991) stated that the morphological data and chaetotaxy of *Echinochasmus* cercaria also show that this genus appears more closely related to the *Psilotrema* (Odhner, 1913) and *Sphaeridiotrema* (Odhner, 1913) than to *Echinostoma*. The Psilostomidae, apart from the absence of a circumoral head-collar armed with spines, closely resemble the Echinostomatidae in their general morphology (Kostadinova 2005b). Species of *Philophthalmus* Looss, 1899 (Echinostomatoidea: Philophthalmidae), whose life cycles include prosobranch snails as first intermediate hosts, formed a well-supported clade in the main clade uniting subfamilies of Echinostomatidae (Fig. 3).

**Figure 4.**
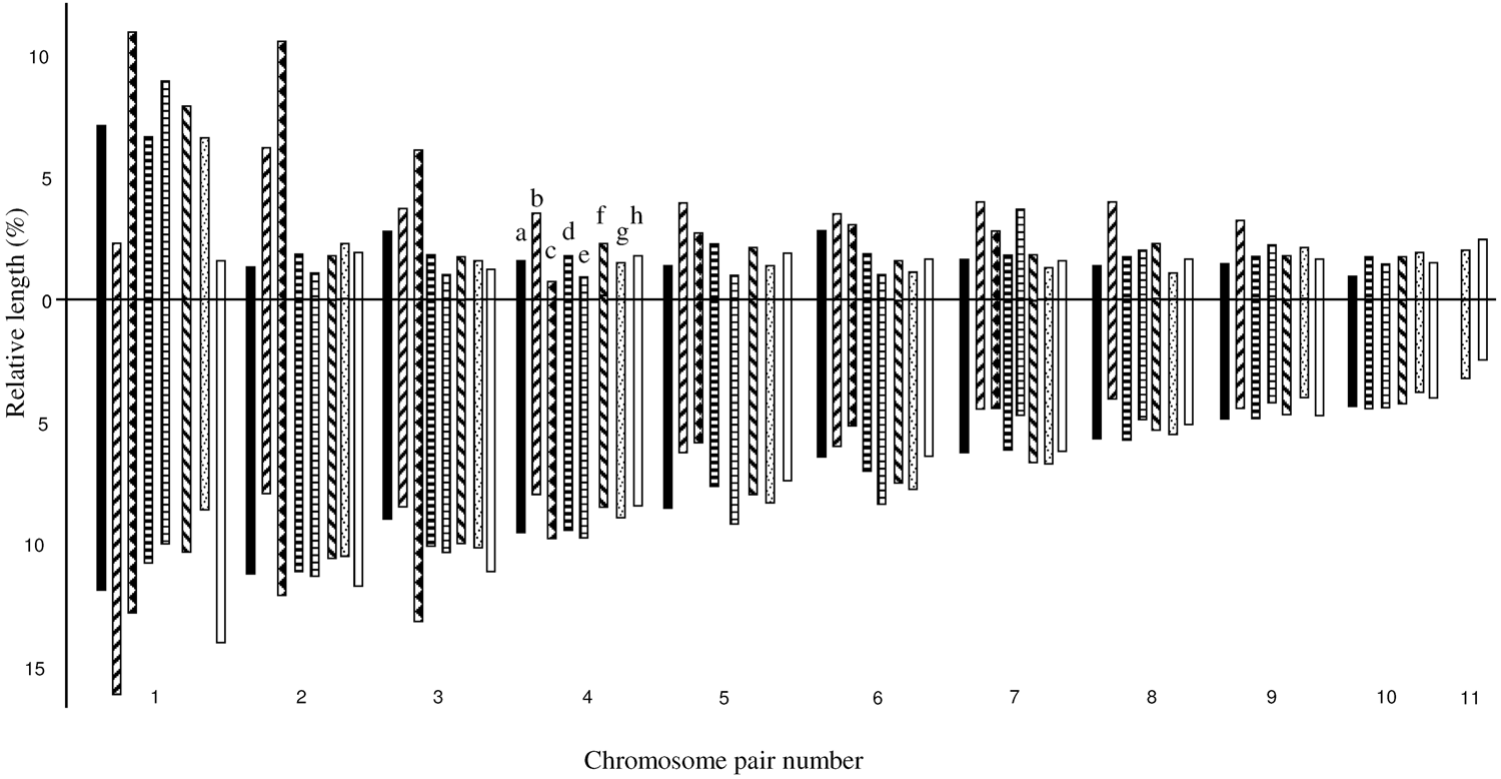
Idiograms representing the haploid chromosome sets. Idiogram representing the haploid sets of eight species: **a** *Echinochasmus* sp. **b** *Episthmium bursicola* **c** *Echinochasmus beleocephalus* **d** *Echinopharyphium aconiatum* **e** *Istmiophora melis* **f** *Hypoderaeum conoideum* **g** *Sphaeridiotrema globulus* **h** *Echinostoma revolutum* **b, c** - data of Baršienė and Kiselienė (1990) **d, e, f, h** data of Baršienė (1993) **g** data of Mutafova (2001).

The chromosome complement of *Echinochasmus* sp. with 2n=22 chromosomes gradually decreasing in size and with one-armed elements prevailing are characteristic for species of type-genus *Echinostoma* (Baršienė 1993; Mutafova 1994). The same chromosome morphology has been reported for species of the genus *Echinopharyphium*, *Neoacanthoparyphium*, *Moliniella*, *Hypoderaeum*, *Isthmiophora* (Echinostomatinae), but in these species the diploid chromosome number is lower, 2n = 20 (see Baršienė 1993 for review, Mutafova 1994). The chromosome number and morphology of *Echinochasmus* sp. resemble the karyotypic data of other representatives of Echinostomatinae (Baršienė 1993). Surprisingly, the other two known karyotypes of species of Echinochasminae are very different from that of *Echinochasmus* sp. Stanevičiūtė et al. 2008. The chromosome number of *Echinochasmus beleocephalus* is 2n=14 and the karyotype consists of three pairs of large biarmed chromosomes and four pairs of smaller homologues. The chromosome set of *Episthmium bursicola* contains 2n=18 and is conspicuous by the presence of a large first pair of subtelocentric elements and the rest of biarmed chromosomes (Baršienė and Kiselienė 1990). The karyotype of Psilostomidae (Echinostomatoidea) – *Psilotrema* sp., *Psilotrema simillimum* (Mühling, 1898) (2n=16), *Psilotrema spiculigerum* (Mühling, 1898) (2n=24) and *Sphaeridiotrema globulus* (2n=14) also vary in their chromosome patterns (Baršienė 1993; Mutafova et al. 1998). Mutafova et al. (2001) studied *Sphaeridiotrema globulus* and found a quite different diploid karyotype (2n=22 instead of 2n=14), with similar characteristic to those found in species of the genus *Echinostoma* 2n=22 and chromosomes of similar relative length; likewise, the centromeric position also varied possibly due to pericentric inversions. A possibility of mistake in the identifications of some species was mentioned by Mutafova et al. (2001). The ideograms of karyotypes of *Echinochasmus* sp. and some discussed species were constructed (Fig. 4) based on the mean values presented in Table 1 and previously published data (Baršienė and Kiselienė 1990, Baršienė 1993, Mutafova et al. 2001). A notable variation in chromosome number and morphology suggest the occurrence of multiple chromosome changes: Robertsonian changes, translocations and pericentric inversions. Chromosome rearrangements in lineage of Echinostomatinae show a karyotypic trend towards reduction in chromosome number, but the main karyotypic changes occurring in a case of speciation in this lineage are multiple pericentric inversions and fit into category of karyotypic orthoselection according to White (1973).

Centric fusions could be a possible mechanism for changes in the chromosomal number in this family and in the other digenean groups (Grossman et al. 1981a,b, Baršienė 1993, Mutafova 1994). Pericentric inversions are also possibly involved in the karyotypic evolution of echinostomatids, since within the group of species with 2n=20 some of them have more biarmed chromosomes than others, while differences in relative length values are not so conspicuous. The notable differences found in the karyotypes of echinochasmine species show the need for further karyological analysis of this family.

The results of this study indicated that the phylogenetic branching of digeneans is related to the nature of their first intermediate host. Moreover, the mode of karyotype evolution correlates with the intermediate host: a remarkable karyotype variation was detected among species parasitizing prosobranch snails, whereas differences among karyotypes of the species parasitizing lymnaeoid pulmonates snails are not significant.

## References

[B1] Baršienė J Kiselienė V (1990) Karyological studies of trematodes within the families Psilostomidae and Echinochasmidae. Helminthologia 27: 99–108.

[B2] Baršienė J (1993) The karyotypes of trematodes. Academia, Vilnius, 370 pp.

[B3] Bowles J Blair D McManus DP (1995) A molecular phylogeny of the human schistosomes. Molecular Phylogenetics and Evolution 4: 103–109. doi: 10.1006/mpev.1995.1011 7663756 10.1006/mpev.1995.1011

[B4] Cribb TH Bray RA Littlewood DTJ (2001) The nature and evolution of the association among digeneans, molluscs and fishes. International Journal for Parasitology 31: 997–1011. doi: 10.1016/j.bbr.2011.03.031 11406146 10.1016/s0020-7519(01)00204-1

[B5] Georgieva S Kostadinova A Skirnisson K (2012) The life-cycle of *Petasiger islandicus* Kostadinova & Skirnisson, 2007 (Digenea: Echinostomatidae) elucidated with the aid of molecular data. Systematic Parasitology 82: 177–183. doi: 10.1007/s11230-012-9354-y 22711507 10.1007/s11230-012-9354-y

[B6] Georgieva S Selbach C Faltýnková A Soldánová M Sures B Skírnisson K Kostadinova A (2013) New cryptic species of the ‘revolutum’ group of Echinostoma (Digenea: Echinostomatidae) revealed by molecular and morphological data. Parasit Vectors 6: . doi: 10.1186/1756-3305-6-64 10.1186/1756-3305-6-64 PMC3605289 23497579

[B7] Grabda-Kazubska B Kiselienė V Bayssade-Dufour Ch (1991) Morphology and chaetotaxy of Echinochasmus sp. cercaria (Trematoda, Echinochasmidae). Annales de Parasitologie Humaine et Comparée 66: 263–268.

[B8] Grabda-Kazubska B Borsuk P Laskowski Z Moné H (1998) A phylogenetic analysis of trematodes of the genus *Echinoparyphium* and related genera based on sequencing of internal transcribed spacer region of rDNA. Acta Parasitologica 43: 116–121.

[B9] Grossman AI Cain GD (1981a) Karyotypes and chromosome morphologies of *Megalodiscus temperatus* and *Philophthalmus gralli*. Journal of Helminthology 55: 71–78.

[B10] Grossman AI Short RB Cain GD (1981b) Karyotype evolution and sex chromosome differentiation in schistosomes (Trematoda, Schistosomatidae). Chromosoma (Berl.) 84: 413–430. doi: 10.1007/BF00286030 7327052 10.1007/BF00286030

[B11] Huffman J Fried B (1990) Echinostoma and echinostomiasis. Advances in Parasitology 29: 215–269. doi: 10.1016/S0065-308X(08)60107-4 2181828 10.1016/s0065-308x(08)60107-4

[B12] Kostadinova A (2005a) Family Echinostomatidae Looss, 1899. In: Jones A Bray RA Gibson DI (Eds) Keys to the Trematoda. Vol. 2 CABI Publishing and The Natural History Museum, Wallingford, 9–64.

[B13] Kostadinova A (2005b) Family Psilostomidae Looss, 1900. In: Jones A Bray RA Gibson DI (Eds) Keys to the Trematoda. Vol. 2 CABI Publishing and The Natural History Museum, Wallingford, 99–118.

[B14] Kostadinova A Gibson D (2000) The systematics of the echinostomes. In: Fried B Graczyk TK (Eds) Echinostomes as Experimental Models of the Biological Research. Kluwer Academic Publishers, Dordrecht, 31–57. doi: 10.1007/978-94-015-9606-0_2

[B15] Kostadinova A Herniou EA Barrett J Littlewood DT (2003) Phylogenetic relationships of *Echinostoma* Rudolphi, 1809 (Digenea: Echinostomatidae) and related genera re-assessed via DNA and morphological analyses. Systematic Parasitology 54: 159–176. doi: 10.1023/A:1022681123340 12652069 10.1023/a:1022681123340

[B16] Kudlai O Stunžėnas V (2013) First description of cercaria of *Stephanoprora pseudoechinata* (Olsson, 1876) (Digenea: Echinostomatidae) using morphological and molecular data. Tropical Medicine and International Health 18 (s1): 230. doi: 10.1111/tmi.12163

[B17] Kudlai O Tkach VV Pulis EE Kostadinova A (2015) Redescription and phylogenetic relationships of *Euparyphium capitaneum* Dietz, 1909, the type-species of Euparyphium Dietz, 1909 (Digenea: Echinostomatidae). Systematic Parasitology 90: 53–65. doi: 10.1007/s11230-014-9533-0 25557747 10.1007/s11230-014-9533-0

[B18] Levan A Fredga K Sandberg AA (1964) Nomenclature for centromeric position on chromosomes. Hereditas 52: 201–220. doi: 10.1111/j.1601-5223.1964.tb01953.x

[B19] Morgan JAT Blair D (1995) Nuclear rDNA ITS sequence variation in the trematode genus *Echinostoma*: an aid to establishing relationships within the 37-collar-spine group. Parasitology 111: 609–615. doi: 10.1017/S003118200007709X 8559594 10.1017/s003118200007709x

[B20] Morgan JAT Blair D (1998a) Mitochondrial ND1 gene sequence used to identify echinostome isolates from Australia and New Zealand. International Journal for Parasitology 28: 493–502. doi: 10.1016/S0020-7519(97)00204-X 9559367 10.1016/s0020-7519(97)00204-x

[B21] Morgan JAT Blair D (1998b) Relative merits of nuclear ribosomal internal transcribed spacers and mitochondrial CO1 and ND1 genes for distinguishing among *Echinostoma* species (Trematoda). Parasitology 116: 289–297. doi: 10.1017/S0031182097002217 9550222 10.1017/s0031182097002217

[B22] Morgan JAT Blair D (2000) Molecular biology of echinostomes. In: Fried B Graczyk TK (Eds) Echinostomes as Experimental Models of the Biological Research. Kluwer Academic Publishers, Dordrecht, 245–266. doi: 10.1007/978-94-015-9606-0_13

[B23] Mutafova T (1994) Karyological studies on some species of the families Echinostomatidae and Plagiorchidae and aspects of chromosome evolution in trematodes. Systematic Parasitology 28: 229–238. doi: 10.1007/BF00009520

[B24] Mutafova T Kanev I Panaiotova M (1998) Chromosomes of *Psilotrema spiculigerum* (Muehling, 1898) Odner, 1913 and *Psilotrema simillimum* (Muehling, 1898) Odner, 1913 (Trematoda: Psilostomidae). Experimental Pathology and Parasitology 1: 22–25.

[B25] Mutafova T Kanev I Panaiotova M (2001) A cytological study of *Sphaeridiotrema globulus* (Rudolphi, 1819) Odhner, 1913. Experimental Pathology and Parasitology 4/5: 24–25.

[B26] Nei M Kumar S (2000) Molecular evolution and phylogenetics. Oxford University Press, New York, 333 pp.

[B27] Noikong W Wongsawad C Chai J-Y Saenphet S Trudgett A (2014) Molecular analysis of Echinostome Metacercariae from their second intermediate host found in a localised geographic region reveals genetic heterogeneity and possible cryptic speciation. PLoS Neglected Tropical Diseases 8(4): . doi: 10.1371/journal.pntd.0002778 10.1371/journal.pntd.0002778 PMC3974680 24699358

[B28] Odening K (1963) Echinostomatoidea, Notocotylata und Cyclocoelida (Trematoda, Digenea, Redionei) aus vögeln des Berliner tierparks. Bijdragen tot de Dierkunde 33: 37–60.

[B29] Olson PD Cribb TH Tkach VV Bray RA Littlewood DT (2003) Phylogeny and classification of the Digenea (Platyhelminthes: Trematoda). International Journal for Parasitology 33: 733–755. doi: 10.1016/S0020-7519(03)00049-3 12814653 10.1016/s0020-7519(03)00049-3

[B30] Olson PD Tkatch VV (2005) Advances and trends in the molecular systematics of the parasitic Platyhelminthes. Advances in Parasitology 60: 165–243. doi: 10.1016/S0065-308X(05)60003-6 16230104 10.1016/S0065-308X(05)60003-6

[B31] Petkevičiūtė R Stanevičiūtė G (1999) Karyotypic characterization of *Apatemon gracilis*. Journal of Helminthology 73: 73–77. doi: 10.1017/S0022149X99000104

[B32] Petkevičiūtė R Stunžėnas V Stanevičiūtė G (2014) Differentiation of European freshwater bucephalids (Digenea: Bucephalidae) based on karyotypes and DNA sequences. Systematic Parasitology 87(2): 199–212. doi: 10.1007/s11230-013-9465-0 24474041 10.1007/s11230-013-9465-0

[B33] Petrie JL Burg EF Cain GD (1996) Molecular characterization of *Echinostoma caproni* and *E. paraensei* by random amplification of polymorphic DNA (RAPD) analysis. Journal of Parasitology 82: 360–362. doi: 10.2307/3284184 8604120

[B34] Posada D (2008) jModelTest: Phylogenetic Model Averaging. Molecular Biology and Evolution 25: 1253–1256. doi: 10.1093/molbev/msn083 18397919 10.1093/molbev/msn083

[B35] Saijuntha W Tantrawatpan C Sithithaworn P Andrews RH Petney TN (2011) Genetic characterization of *Echinostoma revolutum* and *Echinoparyphium recurvatum* (Trematoda: Echinostomatidae) in Thailand and phylogenetic relationships with other isolates inferred by ITS1 sequence. Parasitology Research 10: 751–755. doi 10.1007/s00436-010-2180-8 21120529 10.1007/s00436-010-2180-8

[B36] Saitou N Nei M (1987) The neighbor-joining method: A new method for reconstructing phylogenetic trees. Molecular Biology and Evolution 4: 406–425. 3447015 10.1093/oxfordjournals.molbev.a040454

[B37] Selbach C Soldanova M Georgieva S Kostadinova A Kalbe M Sures B (2014) Morphological and molecular data for larval stages of four species of *Petasiger* Dietz, 1909 (Digenea: Echinostomatidae) with an updated key to the known cercariae from the Palaearctic. Systematic Parasitology 89: 153–166. doi: 10.1007/s11230-014-9513-4 25204601 10.1007/s11230-014-9513-4

[B38] Stanevičiūtė G Petkevičiūtė R Kiselienė V (2008) Digenean parasites in population of prosobranch snail *Lithoglyphus naticoides* with morphological description of *Echinochasmus* sp. cercaria. Ekologija 54: 251–255. doi: 10.6001/ekologija.v54i4.1241

[B39] Stunžėnas V Petkevičiūtė R Stanevičiūtė G (2011) Phylogeny of *Sphaerium solidum* (Bivalvia) based on karyotype and sequences of 16S and ITS1 rDNA. Central European Journal of Biology 6: 105–117. doi: 10.2478/s11535-010-0101-6

[B40] Sudarikov VE Karmanova EM (1977) On validity and structure of the family Echinochasmidae (Odner, 1910). Trudy GELAN 27: 129–41.

[B41] Tamura K Nei M (1993) Estimation of the number of nucleotide substitutions in the control region of mitochondrial DNA in humans and chimpanzees. Molecular Biology and Evolution 10: 512–526. 8336541 10.1093/oxfordjournals.molbev.a040023

[B42] Tamura K Peterson D Peterson N Stecher G Nei M Kumar S (2011) MEGA5: Molecular Evolutionary Genetics Analysis using Maximum Likelihood, Evolutionary Distance, and Maximum Parsimony Methods. Molecular Biology and Evolution 28: 2731–2739. doi: 10.1093/molbev/msr121 21546353 10.1093/molbev/msr121 PMC3203626

[B43] Thompson JD Higgins DG Gibson TJ (1994) CLUSTAL W: improving the sensitivity of progressive multiple sequence alignment through sequence weighting, position-specific gap penalties and weight matrix choice. Nucleic Acids Research 22: 4673–4680. doi: 10.1093/nar/22.22.4673 7984417 10.1093/nar/22.22.4673 PMC308517

[B44] Tkach V Grabda-Kazubska Pawlowski Swiderski Z (1999) Molecular and morphological evidences for close phylogenetic affinities of the genera *Macrodera*, *Leptophallus*, *Metaleptophallus*, and *Paralepoderma* (Digenea, Plagiorchioidea). Acta Parasitologica 44: 17–179.

[B45] White MJD (1973) Animal Cytology and Evolution. 3rd ed Cambridge University Press, Cambridge, 961 pp.

[B46] Wilson WD Johnson PTJ Sutherland DR Mone H Loker ES (2005) A molecular phylogenetic study of the genus *Ribeiroia* (Digenea): trematodes known to cause limb malformations in amphibians. Journal of Parasitology 9: 1040–1045. doi: 10.1645/GE-465R.1 16419746 10.1645/GE-465R.1

[B47] Yamaguti S (1971) Synopsis of digenetic trematodes of vertebrates. Keigaku Publishing Co, Tokyo, 980 pp.

